# Addressing a Yellow Fever Vaccine Shortage — United States, 2016–2017

**DOI:** 10.15585/mmwr.mm6617e2

**Published:** 2017-05-05

**Authors:** Mark D. Gershman, Kristina M. Angelo, Julian Ritchey, David P. Greenberg, Riyadh D. Muhammad, Gary Brunette, Martin S. Cetron, Mark J. Sotir

**Affiliations:** ^1^Division of Global Migration and Quarantine, CDC; ^2^Sanofi Pasteur Inc., Swiftwater, Pennsylvania.

Recent manufacturing problems resulted in a shortage of the only U.S.-licensed yellow fever vaccine. This shortage is expected to lead to a complete depletion of yellow fever vaccine available for the immunization of U.S. travelers by mid-2017. CDC, the Food and Drug Administration (FDA), and Sanofi Pasteur are collaborating to ensure a continuous yellow fever vaccine supply in the United States. As part of this collaboration, Sanofi Pasteur submitted an expanded access investigational new drug (eIND) application to FDA in September 2016 to allow for the importation and use of an alternative yellow fever vaccine manufactured by Sanofi Pasteur France, with safety and efficacy comparable to the U.S.-licensed vaccine; the eIND was accepted by FDA in October 2016. The implementation of this eIND protocol included developing a systematic process for selecting a limited number of clinic sites to provide the vaccine. CDC and Sanofi Pasteur will continue to communicate with the public and other stakeholders, and CDC will provide a list of locations that will be administering the replacement vaccine at a later date.

Yellow fever is an acute viral disease caused by infection with the yellow fever virus, a flavivirus primarily transmitted to humans through the bite of an infected mosquito and endemic to sub-Saharan Africa and tropical South America (*1*). Most infected persons are asymptomatic (*1*). However, the case-fatality ratio is 20%–50% among the approximately 15% of infected persons who develop severe disease (*2*). In recent years, multiple yellow fever outbreaks in Angola, the Democratic Republic of the Congo, and, most recently, Brazil, have underscored the ongoing and substantial global burden of this disease (*3*–*5*).

Yellow fever disease can be prevented by a live-attenuated virus vaccine that produces neutralizing antibodies in 80%–100% of vaccinees by 10 days after vaccination (*2*). For most travelers, only one lifetime dose is necessary (*1*). Vaccination is recommended for international travelers visiting areas with endemic or epidemic yellow fever virus transmission. In addition, proof-of-vaccination is required for entry into certain countries as permitted by the International Health Regulations 2015 (*1*,*6*). To provide proof of vaccination, practitioners at yellow fever vaccination clinics must validate a traveler’s vaccine record using a proof-of-vaccination stamp. CDC has regulatory authority over the designation of U.S. yellow fever vaccination clinics. For nonfederal yellow fever vaccination clinics, this authority to designate is generally delegated and overseen through a collaboration between CDC and state and territorial health departments. CDC maintains the online U.S. Yellow Fever Vaccination Center Registry of these designated clinics.

In 2015, approximately eight million U.S. residents traveled to 42 countries with endemic yellow fever virus transmission (*1*) (Data In, Intelligence Out [https://www.diio.net], unpublished data, 2016). Yellow fever virus can be exported by unimmunized travelers returning to countries where the virus is not endemic. Reports of yellow fever in at least 10 unimmunized returning U.S. and European travelers were recorded during 1970–2013 (*1*). Most recently, yellow fever virus was exported from Angola during the 2016 outbreak to three countries, with resulting local transmission in the Democratic Republic of the Congo (*4*). The Angola outbreak caused 965 confirmed cases from 2015 to 2017 (*4*). The ongoing outbreak in Brazil has resulted in 681 confirmed yellow fever cases from December 2016 through April 25, 2017 (*7*).

In the United States, only one yellow fever vaccine is licensed for use (YF-VAX; Sanofi Pasteur, Swiftwater, PA, 2017); approximately 500,000 doses are distributed annually to vaccinate military and civilian travelers. Approximately two thirds of these doses are distributed among approximately 4,000 civilian clinical sites (Sanofi Pasteur, unpublished data, 2017).

The current YF-VAX supply depletion began in November 2015 (*8*). Sanofi Pasteur was transitioning YF-VAX production from an older to a newer facility set to open in 2018, but a manufacturing complication resulted in the loss of a large number of doses. In response, Sanofi Pasteur instituted YF-VAX ordering restrictions to extend the existing supply while assessing options. In spring 2016, Sanofi Pasteur notified CDC of a probable complete depletion of YF-VAX later in the year. Sanofi Pasteur succeeded in producing additional doses of YF-VAX in late 2016; this additional supply has delayed the anticipated complete depletion until mid-2017 but remains insufficient to cover anticipated demand during the interval between permanent closure of the old facility and the 2018 opening of the new YF-VAX vaccine manufacturing facility.

Concerns about maintaining a continuous U.S. yellow fever vaccine supply, in conjunction with the large yellow fever outbreak that began in Angola, led to discussions among CDC, Sanofi Pasteur, FDA, and the U.S. Department of Defense in spring 2016. Although fractional yellow fever vaccine dosing was discussed, it was deemed a nonviable option based on limited efficacy data. Sanofi Pasteur submitted an eIND application for U.S. importation and civilian use of Stamaril, a yellow fever vaccine manufactured by Sanofi Pasteur France that is not licensed in the United States; the Department of Defense submitted its own eIND application. Stamaril uses the same vaccine substrain 17D-204 as YF-VAX, and has comparable safety and efficacy (*9*). Stamaril has been licensed and distributed in approximately 70 countries worldwide since 1986. Sanofi Pasteur France manufactures both multidose vials for use in global yellow fever outbreak responses and single-dose vials reserved for vaccination of international travelers living outside the United States. Sanofi Pasteur projects that importing Stamaril single-dose vials into the United States under the eIND application will not substantially affect the Stamaril supply intended for global use.

FDA accepted Sanofi Pasteur’s eIND application in October 2016. Implementation of the eIND protocol included a systematic process to select sites where Stamaril will be distributed; this process was important to manage the logistics involved in outreach and training of providers regarding adherence to the eIND protocol and FDA guidance. Sanofi Pasteur, in consultation with CDC, developed a two-tiered scheme for the selection of U.S. clinic sites to be invited to participate in the eIND protocol (Table). The primary goal was to recruit large-volume sites with adequate geographic range. Tier 1 sites were those that ordered at least 250 doses of yellow fever vaccine in 2016. Additional, smaller-volume sites were added to this tier to ensure access to Stamaril in all 50 states, the District of Columbia, and the three U.S. territories (Guam, Puerto Rico, and the U.S. Virgin Islands) with yellow fever vaccination centers. Sites were also added to guarantee vaccine access for civilian U.S. government employees needing yellow fever vaccination for official work-related travel, including critical public health response work. Tier 2 sites included multisite clinical organizations in which the aggregate number of doses ordered from their affiliated sites met the threshold of at least 250 doses in 2016. In these cases, the organization was invited to select one of its clinic sites to participate as a tier 2 site in implementing the Stamaril protocol. As of April 2017, approximately 250 clinics were targeted for inclusion. This is a sizable reduction from the estimated 4,000 civilian clinics currently providing YF-VAX.

**TABLE:** Systematic tiered distribution plan for Stamaril yellow fever vaccine — United States, 2016

Tier	Characteristic	No. of proposed sites
1	Individual sites that ordered at least 250 doses in 2016	193
	Smaller sites to ensure coverage of all 50 states, DC, and U.S. territories	
	Sites that serve non-military U.S. government employees	
2	Sites that are part of a multisite clinical organization whose aggregate number of orders was at least 250 doses in 2016	59
**Total**		**252**

**Abbreviation:** DC = District of Columbia.

The eIND protocol rollout began in April 2017. Sanofi Pasteur and CDC are collaborating to develop an effective communication plan. Sanofi Pasteur is recruiting and communicating with selected sites and will train personnel at participating sites by webinar in April and May 2017.

## Discussion

CDC and Sanofi Pasteur have worked to assure a continuous yellow fever vaccine supply in the United States after the anticipated complete depletion of YF-VAX in mid-2017. As the eIND protocol rollout begins in April, Sanofi Pasteur will coordinate site recruitment and training, and CDC will help to resolve any problems that arise. Although the systematic site selection process for the distribution of Stamaril took into account site volume (giving preference to larger sites) and adequate geographic reach, accessibility difficulties for some international travelers might occur, because of the decrease in the number of clinics nationwide that provide yellow fever vaccination from 4,000 to 250. CDC and Sanofi Pasteur will monitor for critical gaps in vaccine access and collaborate to address any issues, including considering the possibility of recruiting additional clinics to participate as necessary.

CDC will notify state and territorial health department immunization programs about the Stamaril protocol. Information about which clinics will be eligible to receive Stamaril will be available to the public and other stakeholders, and discussed with the Advisory Committee on Immunization Practices. CDC and Sanofi Pasteur continue to monitor the domestic yellow fever vaccine supply and will provide updates to health care providers and the public as new information becomes available.

Updates regarding yellow fever vaccine and the anticipated complete depletion of vaccine stock will be available on CDC’s Travelers’ Health website at https://wwwnc.cdc.gov/travel/ and Sanofi Pasteur’s website at http://www.sanofipasteur.us/vaccines/yellowfevervaccine. Once available, CDC will provide a complete list of clinics where travelers can receive Stamaril at https://wwwnc.cdc.gov/travel/yellow-fever-vaccination-clinics/search.

**Summary**

**What is already known about this topic?**

Effective and safe yellow fever vaccines are available to prevent yellow fever disease among persons traveling to countries with yellow fever virus transmission and to comply with individual country yellow fever vaccination entry requirements; only one yellow fever vaccine (YF-VAX) is currently licensed for use in the United States. Periodic, temporary yellow fever vaccine shortages have occurred in the United States as a result of manufacturing problems, including a manufacturing complication in 2016 that resulted in the loss of a large number of U.S.-licensed yellow fever vaccine doses.

**What is added by this report?**

To avoid a lapse in yellow fever vaccine availability to persons in the U.S. population for whom yellow fever vaccination is indicated, public health officials and private partners collaborated in pursuing an expanded access investigational new drug (eIND) application for the importation of Stamaril yellow fever vaccine into the United States. Stamaril is produced by Sanofi Pasteur, the manufacturer of the U.S.-licensed YF-VAX, and it uses the same vaccine substrain. A systematic, tiered process was developed to select clinics to participate in the eIND protocol, with the goal of reasonable accessibility to yellow fever vaccination for all U.S. residents, while assuring that clinic personnel could be adequately trained to participate in the protocol.

**What are the implications for public health practice?**

Providers need to be aware that there is a yellow fever vaccine shortage and there is a plan for providing safe vaccine at a limited number of clinics until the supply is replenished. Domestic production of yellow fever vaccine in the United States should resume in 2018, and as the eIND protocol is implemented, CDC and Sanofi Pasteur will need to continue to collaborate throughout site recruitment and training, partner to resolve issues that arise, and maintain communication with health care providers and the general public.
