# *Erratum:* Vol. 66, No. 17

**DOI:** 10.15585/mmwr.mm6618a10

**Published:** 2017-05-12

In the report “Addressing a Yellow Fever Vaccine Shortage — United States, 2016–2017,” on page 457, the first sentence of the fourth paragraph should have read “In 2015, **approximately 9.5 million aviation passenger-journeys from the United States to 42 countries with endemic yellow fever virus transmission occurred** (*1*) (Data In, Intelligence Out [https://www.diio.net], unpublished data, 2016).”

